# Hormone guided estrus synchronization using progesterone sponge and PMSG in goats: a cost-effective optimization strategy

**DOI:** 10.3389/fvets.2026.1743340

**Published:** 2026-02-17

**Authors:** Congliang Wang, Xiaoyu Liu, Shutao Shang, Zhihao Wang, Xiayu Yun, Jinwang Liu, Xiaomin Du, Junjun Zhai, Fenghong Wang, Meixia Wang, Jinlian Hua, Haijing Zhu, Lei Qu

**Affiliations:** 1 Shaanxi Provincial Engineering and Technology Research Center of Cashmere Goats, Yulin University, Yulin, Shaanxi, China; 2 College of Veterinary Medicine, Northwest Agriculture & Forestry University, Yangling, Shaanxi, China; 3 College of Advanced Agricultural Sciences, Yulin University, Yulin, Shaanxi, China

**Keywords:** cost, estrus, goat, hormone, progesterone, synchronization

## Abstract

This study aimed to evaluate the efficacy of estrus synchronization protocols in goats and to identify the most cost-effective regimen for intensive farming. The protocols assessed used progesterone (P_4_) sponges or controlled internal drug release (CIDR) devices, with supplemented with pregnant mare serum gonadotropin (PMSG) and prostaglandin (PG), for inducing estrus and pregnancy in goats, and to identify the most cost-effective regimen for intensive farming. During both breeding and non-breeding seasons, does were assigned totreatments: Group I (P_4_ sponge + PMSG), Group II (P_4_ sponge + PMSG + PG), and Group III (CIDR + PMSG + PG). We measured estrus response after device removal and pregnancy rates after artificial insemination (AI) were evaluated and analyzed serum hormone dynamics. Additionally, medication costs were calculated for each protocol. For the most effective protocol, we also determined ovulation timing, performed laparoscopic-assisted AI. Estrus onset occurred earlier in Groups II and III than in Group I during both trial seasons. Nevertheless, overall estrus and pregnancy rates did not differ significantly among the groups (*P* > 0.05), with Group II exhibiting the lowest pregnancy rate. Medication cost per does and per pregnancy were lowest for Group I. Under the Group I protocol, ovulation occurred at 32 h after estrus onset, and laparoscopic-assisted AI yielded a significantly higher pregnancy rate with fresh semen than with chilled semen (*P* < 0.05). Serum estradiol (E_2_) concentration at estrus onset was significantly higher in Group I than in Group II (*P* < 0.05), and luteinizing hormone (LH) levels at estrus onset and ovulation were significantly higher in Groups I and III than in Group II (*P* < 0.05). Serum P_4_, LH, and E_2_ profiles during the estrous cycle followed similar patterns across all groups. In conclusion, there was no significant difference in pregnancy rates between the three estrus synchronization protocols. However, Protocol I demonstrated the lowest per doe and per pregnancy medication costs. Based on cost-effectiveness considerations, the P_4_ sponge + PMSG protocol is recommended for use in intensive goat production systems.

## 1 Introduction

Goat farming is a major economic activity, shifting from small-scale, decentralized operations toward intensive, large-scale systems to meet growing market demand for meat and cashmere (1). However, reliance on natural estrus and mating in does prolongs the lambing interval, reducing farm revenue and increasing costs. Therefore, improving doe herd reproductive rates is a key goal for breeding operations (2). Estrus synchronization offers an effective way to enhance reproductive efficiency in small ruminants. This method uses exogenous hormones to induce synchronized estrus, allows for synchronized artificial insemination (AI) and concentrated lambing, and shortens the herd generation and estrus intervals. Ultimately, it supports batch management and lowers labor and financial inputs (3). The Shaanbei White Cashmere (SBWC) goat, a primary dual-purpose breed for cashmere and meat in northwestern Shaanxi Province, China (4), faces constraints due to seasonal estrus and a predominance of single kidding. Applying estrus synchronization and AI in SBWC goats enables synchronized lambing and simpler management. This approach also reduces the number of bucks needed, extends their productive life, and lowers overall rearing costs (5).

Current estrus synchronization protocols for ruminants often use progesterone impregnated vaginal sponges or controlled internal drug release (CIDR) devices, frequently in combination with prostaglandin (PG), pregnant mare serum gonadotropin (PMSG), and human chorionic gonadotropin (HCG) (6). The physiological basis of estrus synchronization involves the use of exogenous hormones to intervene in the regulatory balance of the hypothalamic-pituitary-ovarian (HPO) axis, thereby aligning follicular development and the estrus onset in a group of animals (7). The main progestogens employed are progesterone (P_4_), fluorogestone acetate (FGA), and medroxyprogesterone acetate (MAP), with P_4_ serving as the primary component in most vaginal sponges and CIDR devices (8). Its core mechanism is negative feedback, which suppresses hypothalamic gonadotropin releasing hormone (GnRH) pulses, thereby reducing pituitary follicle-stimulating hormone (FSH) and luteinizing hormone (LH) secretion (9). This leads to atresia of dominant follicles and blocks new heterogeneous follicular growth. Upon progestogen withdrawal, the negative feedback is lifted, GnRH secretion resumes, and a synchronized release of FSH and LH, triggers a unified wave of follicular development (10). Consequently, the duration of progestogen administration directly impacts synchronization efficacy. Studies have shown that medium and long-term synchronization protocols utilizing P_4_ impregnated sponges or CIDR devices generally yield better results than short-term protocols. Ewes receiving CIDR and PMSG for 14 days showed significantly earlier estrus onset compared with those treated for 6–7 days, along with higher estrus and lambing rates (11, 12). This benefit likely stems from the more thorough clearance of developmentally heterogeneous follicles during extended progesterone treatment, which promotes tighter synchronous recruitment of a new follicular wave under HPO axis control (13). Meta-analyses show that CIDRs and vaginal sponges are equally effective at inducing estrus in does across climates and seasons, though CIDRs often provide superior synchronization. However, the timing of estrus onset remains inconsistent, some studies report a shorter interval with sponges, while others find earlier onset with CIDRs (14). These discrepancies likely reflect differences in breed, season, or handling. In addition, vaginal sponges are more likely than silicone based CIDR devices to cause purulent or hemorrhagic discharge, the co-administration of antibiotics has proven effective in preventing vaginitis (15).

Based on the follicular synchronization established by progesterone, PMSG and PG further regulate follicular development and estrus initiation by targeting ovarian function downstream of the HPO axis (16). PMSG, with its dual FSH and LH activity, is commonly used to synchronize estrus and induce superovulation. After progesterone withdrawal, PMSG works alongside endogenous gonadotropins to stimulate the synchronized growth of recruited follicles, accelerating granulosa cell proliferation and estrogen synthesis (17, 18). Its LH-like activity also promotes androgen production by theca cells, providing precursor for estrogen and further driving follicular development. This coordinated action helps ensure that multiple follicles mature simultaneously, improving estrus synchronization (19, 20). In contrast, PG acts by regressing the corpus luteum, causing a rapid fall in P_4_ (21). This removes the P_4_ mediated negative feedback on the HPO axis, triggering GnRH and gonadotropin release to initiate estrus (22). While simple to administer, PG alone is less reliable for fixed-time AI because the stage of the estrous cycle, and thus luteal status varies among ewes, leading to poor estrus uniformity (23). Therefore, PG is typically combined with a prior P_4_ treatment. P_4_ first synchronizes follicular waves and standardizes luteal development, PG is then given to induce synchronized luteolysis, resulting in tightly synchronized estrus (24).

In addition to PG and PMSG, GnRH and HCG are also important hormones used for estrus synchronization in ruminants. CIDR combined with PMSG and HCG, for instance, can raise the proportion of lambs born in the first 10 days and the overall lambing rate (25). This may be attributed to the LH-like activity of HCG, which supports the post-ovulatory transformation of the follicular wall into a functional corpus luteum, enhancing progesterone secretion and potentially aiding embryo implantation (26). Unlike PMSG, exogenous GnRH administration potently stimulates the anterior pituitary to release LH and FSH in a pulsatile manner. It primarily acts by mimicking the endogenous LH surge to trigger the rupture and ovulation of mature dominant follicles, functioning more as an ovulation inducer than a follicular synchronizing agent (27). Compared to CIDR-PMSG protocols, CIDR-GnRH treatment in ewes does not significantly alter plasma progesterone levels, estrus rate, or pregnancy rate, but it does result in fewer corpora lutea and a lower ovulation rate, and this outcome may be linked to GnRH delaying estrus onset and shortening its duration (28, 29).

This study compared the efficacy of three synchronization protocols, Group I (P_4_ sponge + PMSG), Group II (P_4_ sponge + PMSG + PG), and Group III (CIDR + PMSG + PG), to balance efficiency and cost in goat reproduction. Here, P_4_ sponge and CIDR devices are the most common exogenous progesterone sources, sponges are low-cost, while CIDRs are easier to handle, provide more consistent hormone release, and probable cause less animal irritation. It has been reported that high doses of PMSG may shorten estrus and reduce embryonic potential, 200–300 IU is sufficient for successful fixed-time insemination in goats (30). Therefore, a dose of 250 IU PMSG was selected. PG effectively lyses functional corpora lutea, the literature recommends recommended dose of 0.05–0.1 mg (31, 32). To promote thorough luteal regression, the higher dose of 0.1 mg PG was therefore administered in Groups II and III, with the aim of assessing its combined effect with PMSG to optimize follicular wave synchrony and estrus concentration. High estrus synchronicity after treatment also facilitates AI application. AI techniques include deep vaginal, cervical, and laparoscopic approaches. Unlike vaginal or cervical methods, laparoscopic AI deposits semen directly into the uterine horn or oviduct, shortening the distance and energy required for sperm and oocytes interaction (33). This technique, however, demands accurate prediction of ovulation timing. Currently, no studies have reported ovulation timing in SBWC goats following synchronized estrus. In goat breeding systems, the efficient use of elite bucks genetic resources is essential for accelerating genetic progress. Fresh semen, which representing the optimal biological state, is preferred to maximizing the contribution of superior sires. To overcome geographical limitations, semen preservation through chilling or freezing is necessary for long-distance transport. However, frozen-thawed semen is susceptible to damage from excessive reactive oxygen species (ROS) and lipid peroxidation (LPO), which impair sperm quality and conception rates. In contrast, chilled semen facilitates both cross-regional distribution and the maintenance of high semen quality (34).

Based on previous studies, we hypothesized that (1) the type of progesterone device does not affect the efficacy of estrus synchronization when used with PMSG alone or in combination with PG; (2) the inclusion of PG will induce an earlier estrus onset compared to PMSG alone; (3) and under the optimal protocol, laparoscopic insemination with fresh semen will yield a higher pregnancy rate than with chilled semen. This study aimed to identify a suitable estrus synchronization protocol for intensive goat farming by comparing the efficacy and input costs of three protocols and by evaluating associated reproductive hormone dynamics, thereby contributing to the optimization of the SBWC goat breeding system.

## 2 Materials and methods

### 2.1 Experimental animals

This study was conducted during the breeding (August to October) and non-breeding (April to June) seasons on an intensive goat farm in Yulin City, Shaanxi Province, China. Experimental goats were selected by a random number table method. The procedure was as follows. During the breeding and non-breeding seasons, 165 and 157 female SBWC does that were 2–4 years of age and had a body weight of 40–50 kg were selected, respectively. All selected does had a history of lambing. The animals were randomly divided into three groups, each containing no fewer than 45 individuals. Subsequently, each goat was assigned a unique sequential identification number. Corresponding values from a random number table were then sequentially matched to these numbers. According to the magnitude of these values, the animals were cyclically assigned to Group I, II, and III. The final group sizes were 49, 57, and 59 goats for the breeding season, and 57, 53, and 47 goats for the non-breeding season. Each group was subjected to a different estrus synchronization protocol. During the breeding season, an additional 69 female SBWC does that were also aged 2–4 years and weighed 40–50 kg were randomly selected. Using the same random number table method, they were allocated into two groups consisting of 39 and 30 goats, respectively. These groups then received the estrus synchronization protocol identified as the most effective and cost-efficient, and their ovulation rates were observed and recorded. Semen was collected from 8 SBWC bucks, aged 2–4 years and weighing 60–70 kg. All bucks had prior semen collection experience, had successfully produced offspring via artificial insemination, and exhibited strong libido.

All goats were housed and fed under identical conditions, and they were fed twice daily at 08:00 and 16:00, with water available *ad libitum*. The bucks also received a daily supplement of one fresh egg.

### 2.2 Statistical justification of experimental animal numbers

An *a priori* power analysis was conducted using GPower (v.3.1.9.7, Heinrich-Heine-Universität Düsseldorf, Düsseldorf, Germany), with supplementary confirmation via the resource equation method in SPSS (v.21.0, SPSS Inc., Chicago, IL, USA). Parameter settings were established based on general animal experiment standards and relevant literature (35, 36), statistical power (1-β) = 0.8, significance level (α) = 0.05, and a medium effect size (Cohen's w = 0.3). The calculated sample size was increased by 5%−10% to account for potential losses, such as those due to abnormal estrus.

For the estrus synchronization protocol comparison experiment, the minimum sample size per group for the dichotomous estrus and pregnancy rate outcome was calculated using a chi-square test. Based on an expected estrus rate of 0.8 and a minimum detectable difference of 0.15, the required sample size was 36 does per group. The smallest actual group size used was 47 does, exceeding this minimum. Group sizes were well-balanced ( ≤ 7 % variation). The resource equation method yielded error degrees of freedom of 161 (breeding season) and 153 (non-breeding season), both substantially above the recommended threshold of 10 (37), indicating robustness against individual variation.

For the laparoscopic AI experiment, the required sample size per group to compare pregnancy rates was calculated using a chi-square test for two independent samples. Assuming a pregnancy rate of 0.65, and a target difference of 0.18 (38), the minimum sample size was 25 does per group. The allocated group sizes satisfied this requirement, providing sufficient statistical power.

### 2.3 Estrus synchronization protocols

**Figure 1:**
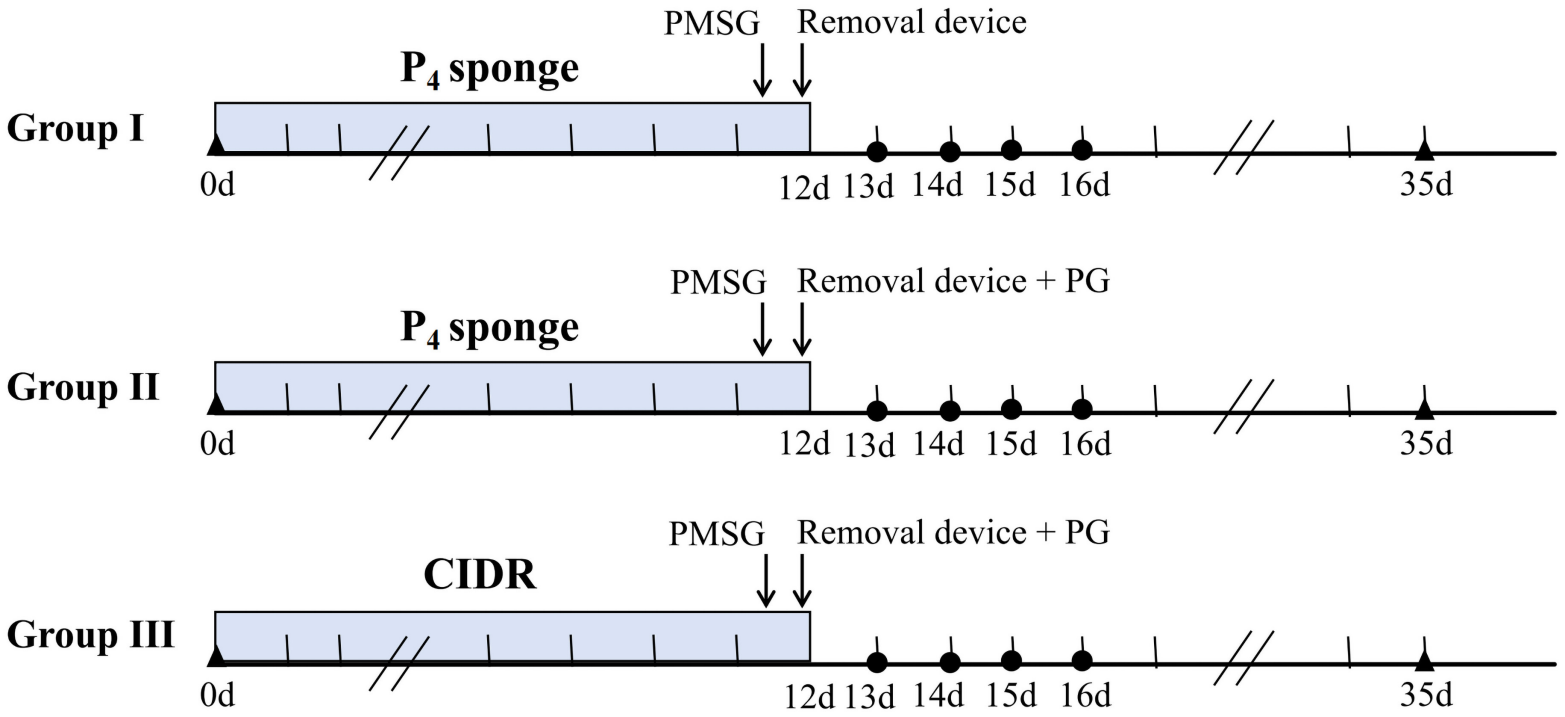
Estrus synchronization protocol for female SBWC does. Blue bars indicate the duration of P_4_ sponge or CIDR treatment. Arrows show the timing of 250 IU PMSG injections, 0.1 mg PG injections and device removal. Circles mark observation periods (0–24 h, 24–48 h, 48–72 h, and 72–96 h post removal). Triangles indicate pregnancy diagnosis time points via B-mode ultrasonography.

Prior to estrus synchronization, transrectal B-mode ultrasonography was performed using a 3.5 MHz transducer (Gandalf Electronic Technology Co., Ltd., Zhengzhou, China) to confirm that all does were non-pregnant. To prevent vaginitis, P_4_ impregnated intravaginal sponges were placed in a container with 3.2 million IU penicillin (Yuanye, #B25911, China) and 4.8 million IU streptomycin (Yuanye, #S17058, China) prior to insertion. The container was gently shaken for 5 min to evenly coat the sponges with the antibiotic mixture. Three estrus synchronization protocols (Group I, II, and III) were implemented. In Groups I and II, a P_4_ impregnated intravaginal sponge (Yanrui Biotechnology Co., Ltd., Yancheng, China) was inserted for 12 days, on the morning of day 12,250 IU PMSG (Ningbo Second Hormone Factory, Zhejiang, China) was administered intramuscularly, and the sponge was removed in the afternoon. Group II additionally received 0.1 mg PG (Ningbo Second Hormone Factory, Ningbo, China) intramuscularly at sponge removal. In Group III, a CIDR insert (Yanrui Biotechnology Co., Ltd., Yancheng, China) was placed intravaginally for 12 days, on the morning of day 12,250 IU PMSG was injected intramuscularly, followed by CIDR removal and an intramuscular injection of 0.1 mg PG in the afternoon. The intravaginal P_4_ sponge and the CIDR contained 50 mg and 300 mg of P_4_, respectively. The specific protocols and timelines are summarized in Figure 1.

### 2.4 Estrus detection and intracervical AI

Estrus observation began after the the synchronization protocol was completed and was performed at 4 h intervals. Estrus was defined as the period during which a doe stood still and accepted mounting by a buck while wagging her tail (Supplementary Figure 1A). Estrus was considered to have ended when mounting was no longer accepted. To evaluate the efficiency of each protocol, the number of does exhibiting estrus was recorded within the intervals of 0–24 h, 24–48 h, 48–72 h, and 72–96 h after sponge or CIDR removal.

Semen was collected from 8 bucks using an artificial vagina. Sperm motility and density were evaluated using a phase-contrast microscope. Ejaculates meeting the following criteria were used for AI: volume 0.8–1.5 ml, motility ≥ 85 %, and concentration ≥ 2 × 10^9^ sperm/ml. Collections from each buck were separated by at least one day to maintain semen quality. Qualified samples from each collection were pooled and homogenized in sterilized centrifuge tubes to reduce individual variation. The pooled semen was diluted with pre-warmed 0.9 % saline (Kelun Pharmaceutical Co., Ltd., Chengdu, China) to a final sperm density of approximately 1 × 108 sperm/ml.

Intracervical AI was performed 12 h after the estrus onset and repeated 12 h later. All procedures were conducted by the same technician using an AI instrument with a light source (Xiaofuzhu Animal Husbandry Technology Co., Ltd., Zhengzhou, China). A volume of 0.2 mL of diluted semen was slowly deposited into the cervical canal. Pregnancy was confirmed 30 days after AI using a 3.5 MHz rectal B-mode ultrasound scanner, and the pregnancy rate was calculated. Pregnancy diagnosis was performed with reference to Supplementary Figure 1B.

### 2.5 Calculation of estrus synchronization drug costs

Pharmaceutical costs in this study were based on market prices at the time of the research. A bag of P_4_ sponges (400 CNY) and a CIDR device (750 CNY) each supplied 50 does, resulting in per-doe costs of 8 CNY and 15 CNY, respectively. One box of PMSG (100 CNY) contained five 1,000 IU vials and could treat 20 does, cequaling 5 CNY per doe. A box of PG (50 CNY) included ten 0.2 mg vials, also covering 20 does, for a unit cost of 2.5 CNY per doe. Due to minimal usage, penicillin and streptomycin were excluded from the cost analysis.

### 2.6 Observation of ovulation and laparoscopy-assisted uterine horn AI

Sixty nine SBWC goats does were randomly assigned to two groups. All animals underwent estrus synchronization using the Group I protocol, which was selected based on prior experimental results. Estrus was monitored at 4 h intervals. The Interval from sponge removal to estrus onset, estrus duration, and interval from estrus onset to ovulation were recorded. Ovulation timing was assessed beginning 24 h after estrus onset, with examinations performed every 2 h. For this procedure, does were anesthetized by intravenous injection of 0.4 ml of anesthetic (Bite Biotechnology Research Institute Co., Ltd., Changsha, China). After anesthesia and abdominal hair removal, does were placed in dorsal recumbency on a surgical rack fixed at a 45 °. Using a laparoscope (Baiqin Medical Technology Co., Ltd., Zhengzhou, China) with atraumatic grasping forceps, the oviductal fimbriae were gently retracted to fully expose the ovary, and the precise ovulation time was documented.

Individual male differ substantially in cryotolerance (39). Using pooled semen would introduce confounding, obscuring whether outcomes were due to preservation method or bucks identity. Therefore, semen was collected from 1 buck. Fresh semen was collected and diluted according to an established protocol. To prepare the refrigerated extender, the following components were accurately weighed and combined in a beaker: 4.54 g Tris (Solarbio, #T8060, China), 0.2 g glucose (Solarbio, #G8151, China), 4.6 g fructose (Solarbio, #F8100, China), 1.5 g EDTA (Solarbio, #E8040, China), 2.38 g sodium citrate (Solarbio, #SS6100, China), 100,000 IU penicillin sodium, and 100,000 IU streptomycin sulfate. These compounds were dissolved in 80 ml of double-distilled water under continuous stirring. The solution was filtered through a 0.22 μm filter into a sterilized glass bottle and brought to a final volume of 100 ml. Sterilization was achieved byincubation in a 56 °C water bath for 30 min. For use, 8 ml of the base extender was combined with 2 ml of egg yolk. The collected fresh semen was diluted with this mixture to a final concentration of 1 × 10^8^ sperm/ml. The diluted semen was transferred to a 15 ml centrifuge tube, wrapped in ten layers of gauze, and stored at 4 °C. Only samples showing > 40 % motility after rewarming in a 37 °C water bath were used for laparoscopy-assisted AI. After laparoscopic confirmation of ovulation, the corresponding uterine horn was gently exteriorized using atraumatic forceps. A sterilized paper clip was used to create a small puncture at the anterior end, through which 0.2 ml of either fresh or chilled semen was injected. Following anesthetic recovery, does were returned to their pens and maintained under standard feeding conditions. Pregnancy was diagnosed 30 days later via B-mode ultrasonography.

### 2.7 Determination of serum LH, P_4_, and E_2_ concentrations

Previous studies indicate that different estrus synchronization treatments do not significantly affect the timing of the LH surge or ovulation in goats (40). During the breeding season, blood samples were collected via jugular venipuncture from does in the three treatment groups at the following time points: sponge removal, estrus onset, ovulation onset (32 h post-estrus), and estrus end. Serum was separated (*n* = 5 per group). Concentrations of P_4_, LH, and E_2_ were measured using enzyme-linked immunosorbent assay (ELISA) kits (Enzyme Tech Biotechnology Co., Ltd., Shanghai, China); detection ranges were 35–1600 pmol/L for P_4_, 0.6–35 ng/L for LH, and 1.5–80 ng/L for E_2_. Absorbance was read at 450 nm using a Varioskan LUX multimode microplate reader (Thermo Fisher Scientific, MA, USA). Standard curves were plotted from known concentrations and corresponding OD values, and sample concentrations were calculated accordingly. Intra and inter-assay coefficients of variation were 5.4 % and 7.5 %, respectively.

### 2.8 Data statistics and analysis

All statistical analyses were performed using SPSS (version 26.0, SPSS Inc., Chicago, IL, USA). The normality of the data was assessed with the Shapiro-Wilk test, and the homogeneity of variances was evaluated using Levene's test. Estrus and pregnancy rates were compared across treatment groups using the chi-square test, with risk ratios (RR) and 95 % confidence intervals (CI) calculated. Serum concentrations of P_4_, LH, and E_2_ were analyzed by one-way analysis of variance (ANOVA) followed by LSD *post hoc* tests. A *P* < 0.05 was considered statistically significant.

## 3 Results

### 3.1 Estrus percentage of does at different intervals following estrus synchronization during breeding and non-breeding seasons

No P_4_ sponges or CIDR were lost during the experiment. The interval from 0–96 h after device removal was regarded as the effective estrus synchronization window. During the breeding season (Table 1), estrus rates differed significantly among the three Group within the 0–24 h and 24–48 h after treatment (*P* < 0.05), but not during later intervals (*P* > 0.05). In the non-breeding season, significant differences were also observed at 0–24 h, 24–48 h, and 48–72 h (*P* < 0.05). In both seasons, Group I showed a significantly higher estrus rate at 24–48 h than at other time points (*P* < 0.05), while Group III reached its highest rate within 0–24 h (*P* < 0.05). In contrast, Group II exhibited the highest estrus rate at 0–24 h during the breeding season, and at both 0–24 h and 24–48 h during the non-breeding season (*P* < 0.05).

**Table 1:** Distribution of estrus concentration time following synchronization treatment with different protocols during breeding and non-breeding seasons (%).

**Time periods**	**Breeding season**			**Non-Breeding season**		
	**Percentage of estrous ewes**					
	**Group I**	**Group II**	**Group III**	**Group I**	**Group II**	**Group III**
0–24 h	0 (0/49)	**78.9**^**a**^ **(45/57)**	**76.3**^**a**^ **(45/59)**	0 (0/57)	**37.7**^**Ba**^ **(20/53)**	**72.3**^**Aa**^ **(34/47)**
24–48 h	**84.6**^**Aa**^ **(44/49)**	5.3^Cb^ (3/57)	16.9^Bb^ (10/59)	**61.4**^**Aa**^ **(35/57)**	**39.6**^**Ba**^ **(21/53)**	4.3^Cb^ (2/47)
48–72 h	3.8^b^ (2/49)	1.8^b^ (1/57)	0 (0/59)	15.8^Ab^ (9/57)	0.00 (0/53)	2.1^Bb^ (1/47)
72–96 h	1.9^b^ (1/49)	0 (0/57)	0 (0/59)	1.8^c^ (1/57)	1.9^b^ (1/53)	0 (0/47)

Group I, P_4_ sponge + PMSG; Group II, P_4_ sponge + PMSG + PG; Group III, CIDR + PMSG + PG. Different uppercase letters as superscripts within the same row denote significant differences in the estrus rate among protocols during a same period (*P* < 0.05). Different lowercase letters as superscripts within the same column indicate significant differences in the estrus rate for a single protocol across different periods (*P* < 0.05).

The bolded values indicate the most significant estrus or pregnancy rate.

### 3.2 Estrus synchronization efficacy in does during breeding and non-breeding seasons

The overall estrus and pregnancy outcomes following estrus synchronization in does during both seasons are shown in Table 2. During the breeding season, Group I showed the highest total estrus and pregnancy rates, but not differ significantly from those in Groups II and III (*P* > 0.05). In the non-breeding season, no significant differences in total estrus or pregnancy rates were found across groups (*P* > 0.05).

**Table 2:** Overall estrus rate and pregnancy rate following synchronization treatment with different protocols during breeding and non-breeding seasons (%).

**Periods**	**Variables**	**Treatment protocol**		
		**Group I**	**Group II**	**Group III**
Breeding season	No. of does (*n*)	49	57	59
	Estrous response (%)	95.9 (47/49)	86 (49/57)	93.2 (55/59)
	Pregnancy (%)	72.3 (34/47)	65.3 (32/49)	70.9 (39/55)
	RR (95% CI)	1.00	0.930 (0.544~3.142)	0.980 (0.484–2.684)
Non-breeding season	No. of does (*n*)	57	53	47
	Estrous response (%)	78.9 (45/57)	79.0 (42/53)	78.7 (37/47)
	Pregnancy (%)	64.4 (29/45)	59.5 (25/42)	64.9 (24/37)
	RR (95% CI)	1.00	0.92 (0.518–2.934)	1.01 (0.395–2.439)

Group I, P_4_ sponge + PMSG; Group II, P_4_ sponge + PMSG + PG; Group III, CIDR + PMSG + PG; RR, risk ratio; CI, 95 % confidence intervals.

### 3.3 Cost of estrus synchronization medications

**Figure 2:**
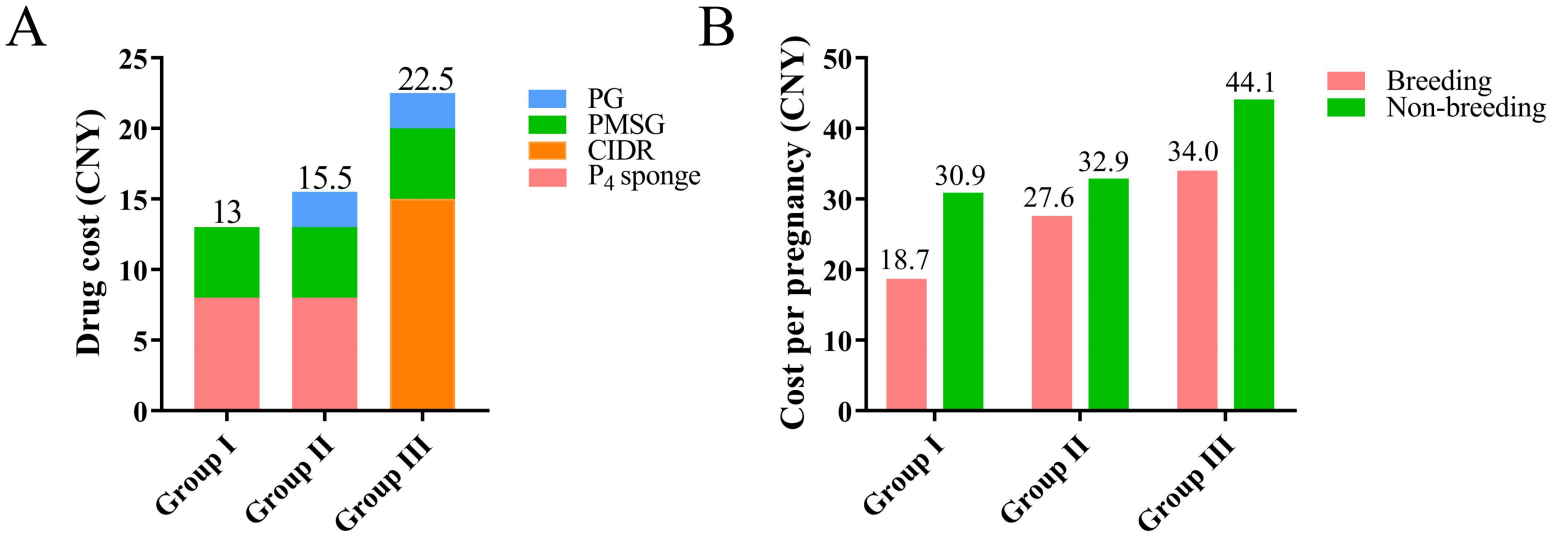
Drug costs of the estrus synchronization protocols. **(A)** Cost of estrus synchronization drugs per does, **(B)** Drug cost per pregnancy for the three protocols. Groups: I (P_4_ sponge + PMSG), II (P_4_ sponge + PMSG + PG), III (CIDR + PMSG + PG).

The cost per does and per pregnancy for the three protocols is presented in Figure 2. The Group III protocol was the most expensive at 22.5 CNY, while Group I was the least expensive at 13 CNY. Group II had a moderate cost of 15.5 CNY, which was slightly higher than that of Group I (Figure 2A). Group I consistently showed the lowest cost per pregnancy during both the breeding and non-breeding seasons, at 18.7 and 30.9 CNY, respectively. In contrast, Group III was the most expensive, with costs of 34.0 and 44.1 CNY for the two seasons (Figure 2B).

### 3.4 Ovulation time and laparoscope-assisted uterine horn AI

Based on efficacy and cost, the Group I protocol was selected as the optimal estrus synchronization regimen. During the breeding season, does in both groups were synchronized using the Group I protocol, and ovulation was monitored by laparoscopy. The results (Table 3) showed that the estrus rates no significant difference between them (*P* > 0.05). The intervals from sponge removal to estrus onset, estrus duration, and the intervals from estrus onset to ovulation, none of these parameters differed significantly (*P* > 0.05). Laparoscope-assisted AI into the uterine horn showed that the pregnancy rate with fresh semen was significantly higher than with chilled semen (*P* < 0.05).

**Table 3:** Interval from sponge removal to estrus onset, estrus duration, and interval from estrus onset to ovulation following synchronization treatment with Group I protocol during the breeding season, and laparoscopy assisted AI with fresh and chilled semen.

**Group**	**No. of does (*n*)**	**Estrous response (%)**	**Pregnancy (%)**	**Interval from sponge removal to estrus onset/h**	**Estrus duration/h**	**Interval from estrus onset to ovulation/h**	**RR (95% CI)**
Fresh	39	94.9 (37/39)	**95**^**a**^ **(35/37)**	34.6 ± 1.8	39.1 ± 0.7	31.4 ± 0.6	1.00
chilled	30	90 (27/30)	63^b^ (17/27)	34.9 ± 0.7	39.8 ± 0.9	32.3 ± 0.8	0.665 (0.493–2.027)

Data labeled with different letters in the same column indicate significant differences in pregnancy rates (*P* < 0.05); RR, risk ratio; CI, 95 % confidence intervals.

The bolded values indicate the most significant estrus or pregnancy rate.

### 3.5 Comparison of reproductive hormones in does treated with different estrus synchronization protocols

**Figure 3:**
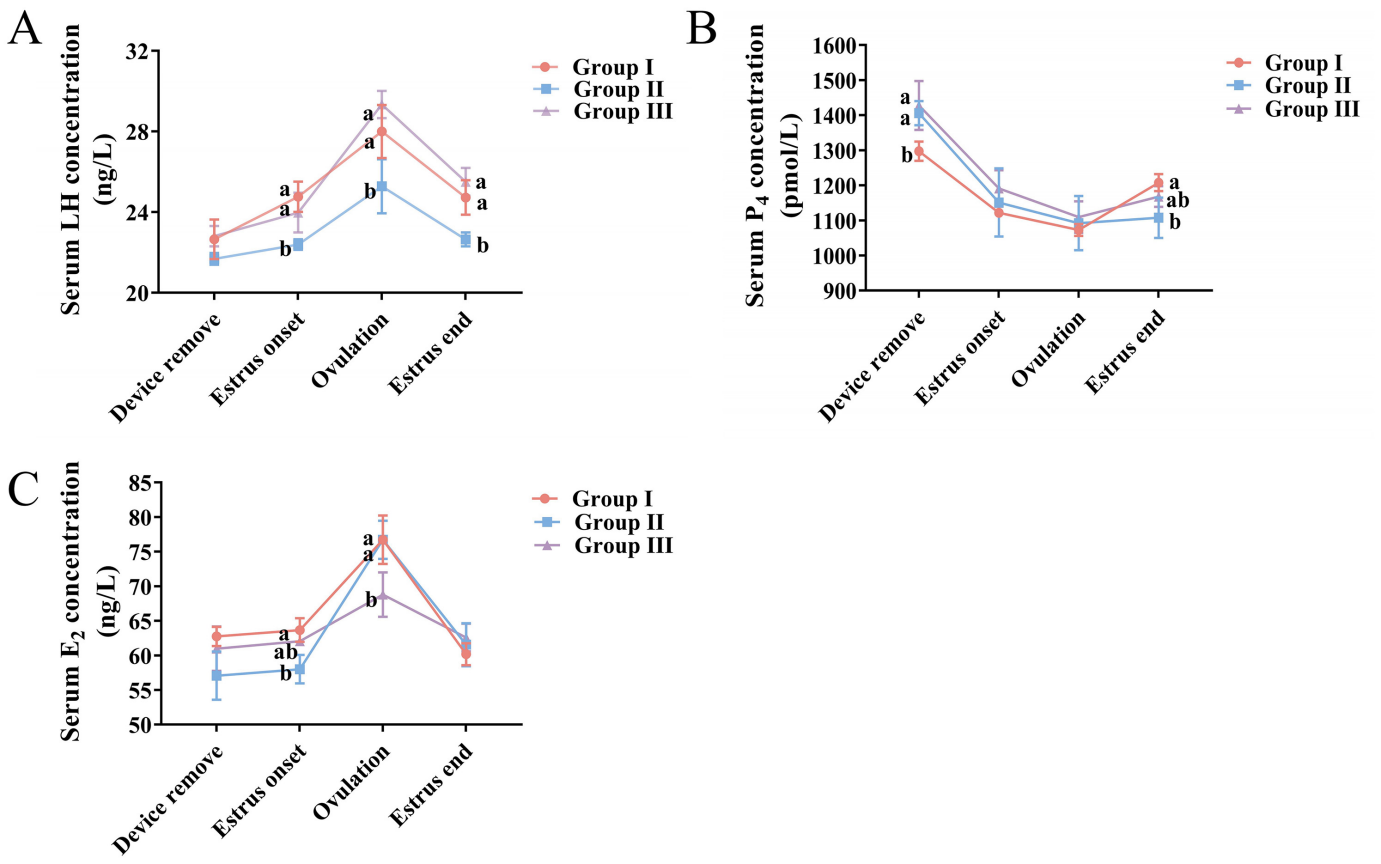
Serum hormone concentrations of LH, P_4_, E_2_ in does under different synchronization protocols. **(A)** LH, **(B)** P_4_, and **(C)** E_2_ levels across groups at four time points: after device removal, estrus onset, ovulation, and estrus end. Groups: I (P_4_ sponge + PMSG), II (P_4_ sponge + PMSG + PG), III (CIDR + PMSG + PG). Different superscript letters indicate significant differences (*P* < 0.05).

Figure 3 shows reproductive hormone concentrations in does at different time points after estrus synchronization. At the estrus onset, ovulation, and the estrus end, LH concentrations were significantly higher in Groups I and III than in Group II (*P* < 0.05). Overall, LH levels first rose and then declined (Figure 3A). After device removal, the P_4_ concentration in Groups II and III was significantly higher than in Group I (*P* < 0.05). By the estrus end, the P_4_ in Group I had become significantly higher than in Group II (*P* < 0.05). P_4_ levels overall first decreased and then increased (Figure 3B). No significant differences in E_2_ concentration were seen among the three groups immediately after device removal or at estrus end (*P* > 0.05). However, at the estrus onset, E_2_ in Group I was significantly higher than in Group II, and at ovulation, E_2_ in Groups I and II was significantly higher than in Group III (*P* < 0.05). E_2_ concentration across protocols showed an initial increase followed by a decrease (Figure 3C).

## 4 Discussion

Long-term progesterone-based synchronization protocols (11–14 days) is known to advance estrus onset and significantly improve pregnancy rates in ewes (11), an effect that appears independent of the progesterone device type (12), Accordingly, a 12 day synchronization period was used for groups in this study. Estrus typically began within 48 h after treatment ended (29), which aligns with our findings: all three protocols induced estrus within this period. However, does in Groups II and III, which received PG estrus earlier (0–24 h) started than those in Group I (P_4_ sponge + PMSG), consistent with earlier reports (41). PG rapidly lyses the corpus luteum, promptly inducing estrus (21). Meanwhile, the estrus rate in Group I during 24–48 h was significantly higher than in the other groups. Group III achieved its highest estrus rate within 0–24 h in both seasons. Although Group II peak estrus period varied seasonally, its overall estrus rate remained stable. Together, all three protocols effectively induced estrus in does across reproductive stages.

Sustained PMSG stimulation can disrupt ovulation synchrony, leading to the simultaneous presence of corpora lutea and follicles in doe ovaries and impairing the expression of functional genes such as Aquaporin 3 (*AQP3*) (42). In a study by Sun et al. (43) a single 300 IU PMSG injection resulted in an estrus rate of 95.8 % and a pregnancy rate of 68.18 %. However, three injections of 300 IU PMSG significantly lowered both rates. These findings align with our results, suggesting that a PMSG dose of 250–300 IU is generally suitable for inducing estrus while maintaining satisfactory pregnancy outcomes in goats. Due to its long half-life, excessive PMSG causes persistent ovarian stimulation and elevate PMSG antibody concentrations in follicles and plasma (43). The antibody binds PMSG, forming a complex that still activates follicular FSH/LH receptors, and this results in temporal disparities in gonadotropin response follicles, thereby inducing developmental heterogeneity (44). Excess antibody can also suppress or delay the preovulatory LH surge, preventing synchronous final maturation of some follicles (45). These immature follicles continue secreting E_2_ under residual PMSG stimulation. The elevated E_2_ further exacerbates maturational asynchrony, leading to a prolonged and disordered ovulation window (46). Severe manifestations may include ovarian cyst formation and compromised fertilization and implantation, ultimately reducing the efficacy of estrus synchronization and overall reproductive performance (47). Although estrus efficiency differed significantly among the protocols during both breeding and non-breeding seasons, the overall estrus and pregnancy rates did not. This outcome likely reflects the uniform 12 day treatment duration and the appropriate PMSG dosage used in all three protocols. This approach ensured effective synchronization of the follicular wave, thereby offsetting any differential effects arising from the use of distinct progestogen carriers and hormonal combinations (11). Interestingly, the Group II consistently showed the lowest pregnancy rate, likely because PG alters hormonal dynamics. By inducing rapid luteolysis, PG increases plasma estradiol and disrupts the endogenous FSH rhythm, which can cause poorly timed follicle rupture and luteal insufficiency, impairing implantation (48). Additionally, PG injection reduces tubal-directed uterine contractions, limiting sperm transport. Combined with potential mild vaginitis from the sponge, these factors may also contribute to the lower pregnancy rate (49, 50).

It is well documented that the use of progesterone sponges increases the risk of vaginitis and alters vaginal microbiota in ewes (51). It can induce vaginal epithelial hyperplasia and hypertrophy, elevating local counts of neutrophils, macrophages, and red blood cells (52). Sponge expulsion is also a common risk. Collectively, these changes reduce the sexual attractiveness of ewes (53), and when combined with increased bacterial load and purulent secretions, may impair sperm function and survival after AI (54). Consequently, adjunct antibiotic therapy is commonly used to lower vaginitis rates (55), and while effective against purulent discharge, it may not enhance pregnancy outcomes (56). Alternatively, CIDR devices probable reduce vaginitis but increase cost (14). In our study, progesterone sponges were combined with antibiotics to prevent vaginitis, and no sponge expulsion occurred. Estrus and pregnancy rates in these groups were not significantly lower than in CIDR-treated does. This aligns with findings that antibiotic administration, while not completely preventing vaginitis, does not compromise fertility (57). Furthermore, a comparative analysis of the advantages and disadvantages of the three protocols was conducted (Table 4). The defining characteristics are the progesterone delivery method and the use of PG, which correlate with their suitability for farms of different scales. The principal advantage of Group I is its low cost, making it applicable for small-scale or resource-constrained operations. Group II, which incorporates PG to regress the corpus luteum, provides a quicker estrus onset at a relatively low incremental cost. A common limitation of both Group I and II is their reduced effectiveness during the non-breeding season, coupled with an increased risk of vaginitis (51). Group III, conversely, delivers operational ease and improved animal comfort by maintaining a stable progesterone level, resulting in better synchronization. Nevertheless, its high per-unit cost is a significant barrier, thus constraining its utility in extensive production systems (58). Beyond reproductive performance, the cost of synchronization protocols is a critical consideration for farm management. Higher medication expenses did not correspond to significant improvements in estrus response or reproductive outcomes (59). Considering both cost and efficacy, the Group I protocol is recommended as the optimal estrus synchronization regimen for does.

**Table 4:** Comparison of the advantages and disadvantages of three estrus synchronization protocols.

**Protocols**	**Advantages**	**Disadvantages**
Group I	1. The lowest cost; 2. The simplest operation, with a low technical threshold; 3. Eliminates the need for a PG injection, thereby reducing stress	1. Slower initiation and longer duration of estrus; 2. Carries a risk of inducing mild vaginitis
Group II	1. Achieves a more concentrated and earlier estrus; 2. Provides superior synchronization compared to Group I; 3. Is less costly than Group III	1. Is more costly than Group I; 2. The additional PG injection adds procedural complexity and animal stress; 3. PG administration may induce short-term discomfort in individual animals; 4. Carries a risk of inducing mild vaginitis
Group III	1. Offers more stable progesterone release, lowering the risk of vaginal issues; 2. Delivers the highest synchronization efficacy; 3. Operation is convenient, with easy insertion and removal	1. Incurs a higher single-use cost; 2. Demands higher operator proficiency; 3. Has lower cost-effectiveness, hindering use in economically disadvantaged regions

Compared with traditional cervical AI, laparoscopy allows direct observation of ovulation and shortens the distance sperm must travel, improving conception rates and enabling timely assessment of ovarian and uterine health. This method also helps identify and remove does with ovarian hypoplasia or cysts (33). Given the favorable outcomes and cost-effectiveness of the Group I protocol, laparoscopy was used to determine ovulation timing. Ovulation occurred approximately 32 h after estrus in both groups, with no significant differences in synchronized estrus rates, the interval from sponge removal to estrus onset, or estrus duration. These results confirm that the Group I protocol reliably induces estrus and ovulation in does. However, the pregnancy rate after laparoscopy-assisted AI exceeded 90 % with fresh semen, significantly higher than with chilled semen. Previous studies show that using yolk alone as a cryoprotectant can reduce sperm motility by > 45 % and markedly impair plasma membrane integrity and antioxidant capacity (60), which likely explains the lower pregnancy rates with chilled semen. In summary, the Group I protocol effectively induces estrus and ovulation, and performing AI within about 32 h after estrus significantly increases the pregnancy rate in does.

Our studies established that ovulation in does treated with the Group I protocol occurs around 32 h after estrus, with no significant link between LH surge timing, ovulation time, and the synchronization protocol used (28). Therefore, serum concentrations of LH, P_4_, and E_2_ were measured at four time points: after sponge removal, at estrus onset, at ovulation (32 h after estrus onset), and at the estrus end. Overall, P_4_ levels decreased rapidly and then increased gradually across all protocols, while LH and E_2_ levels first rose slowly and later declined. Specifically, sponge/CIDR removal ended the exogenous P_4_ supply, causing P_4_ to fall rapidly. This drop reduced negative feedback on the hypothalamic-pituitary axis (10). Under stimulation of pituitary FSH and exogenous PMSG, follicles were synchronously recruited and development (17). During this process, granulosa cells produced large amounts of E_2_, which directly induced estrous behavior (61). The LH surge then triggers final follicular maturation and subsequent ovulation (19), and low peri-ovulatory P_4_ levels initiate this LH surge (62). After ovulation, LH, P_4_, and E_2_ levels gradually returned to baseline. In this study, at ovulation, LH was significantly higher in Groups I and III than in Group II, while the E_2_ at estrus onset was higher in Group I than in Group II. These hormonal differences likely explain the lowest estrus and pregnancy rates in Group II, since higher E_2_ and LH levels promote effective estrus behavior and ovulation (63).

Although this study identified a suitable estrus synchronization protocol for intensive goat production and demonstrated the efficacy of laparoscopic AI with fresh and chilled semen under optimal conditions, providing practical data for the industry, it has several limitations. First, the work used only SBWC goats. The reproductive physiology of this breed, including its hormonal sensitivity, may differ from others, limiting the direct extrapolation of conclusions to multi-breed systems. Second, all trials were conducted on a single farm with uniform management and operators. In practice, variability in facilities, management, and technical skill between farms could influence protocol outcomes. Third, we applied a fixed PMSG dose (250 IU) based on literature, without testing a dose gradient. The optimal PMSG dose likely interacts with body weight, age, parity, and season; thus, our study could not determine dose response effects or identify breed or state specific optima. Finally, a comparison of laparoscopic-assisted AI using fresh and chilled semen was conducted using semen from only one buck. This design effectively controlled for inter-bucks variation, clearly demonstrated the pregnancy outcomes for both semen types, and allowed the observed difference to be attributed to the preservation method. However, because individual bucks vary in their semen tolerance to cryopreservation, the results may not be directly generalizable to all bucks. In addition, laparoscopic AI with frozen semen under the optimal protocol was not validated, restricting the protocol applicability and failing to provide a technical reference for frozen semen use. Future studies should test these findings in multiple breeds, optimize hormonal doses, compare AI outcomes across semen preservation types, including frozen, based on a larger cohort of bucks, and perform detailed long-term cost benefit analyses.

## 5 Conclusion

In summary, Protocol I (P4 sponge + PMSG) demonstrated efficacy in synchronizing estrus, supporting ovulation, and achieving pregnancy via AI. An evaluation of the drug treatment cost per ewe and per pregnancy for the three protocols showed that Group I had the lowest cost per does, at only 13 CNY. It also had the lowest cost per pregnancy in both seasons, with costs of 18.7 CNY in the breeding season and 30.9 CNY in the non-breeding season. Therefore, considering the induced estrus rate, pregnancy rate, and medication costs, Group I is recommended as the optimal synchronization protocol for SBWC goats, provides data support for the intensive farming and breeding production of goats.

## Data Availability

The original contributions presented in the study are included in the article/supplementary material, further inquiries can be directed to the corresponding author/s.

## Supplementary material

SUPPLEMENTARY FIGURE 1 Estrus detection and ultrasonic pregnancy diagnosis in does. **(A)** Does in estrus being mounted by a buck. **(B)** Pregnancy confirmed via ultrasonography 30 days after AI, with the early embryonic vesicle marked by a red circle.
