# Supplementary figures and images for: Hormone guided estrus synchronization using progesterone sponge and PMSG in goats: a cost-effective optimization strategy
Source: Front Vet Sci. 2026 Feb 17;13:1743340. doi: 10.3389/fvets.2026.1743340 (PMC12953086; doi:10.3389/fvets.2026.1743340)

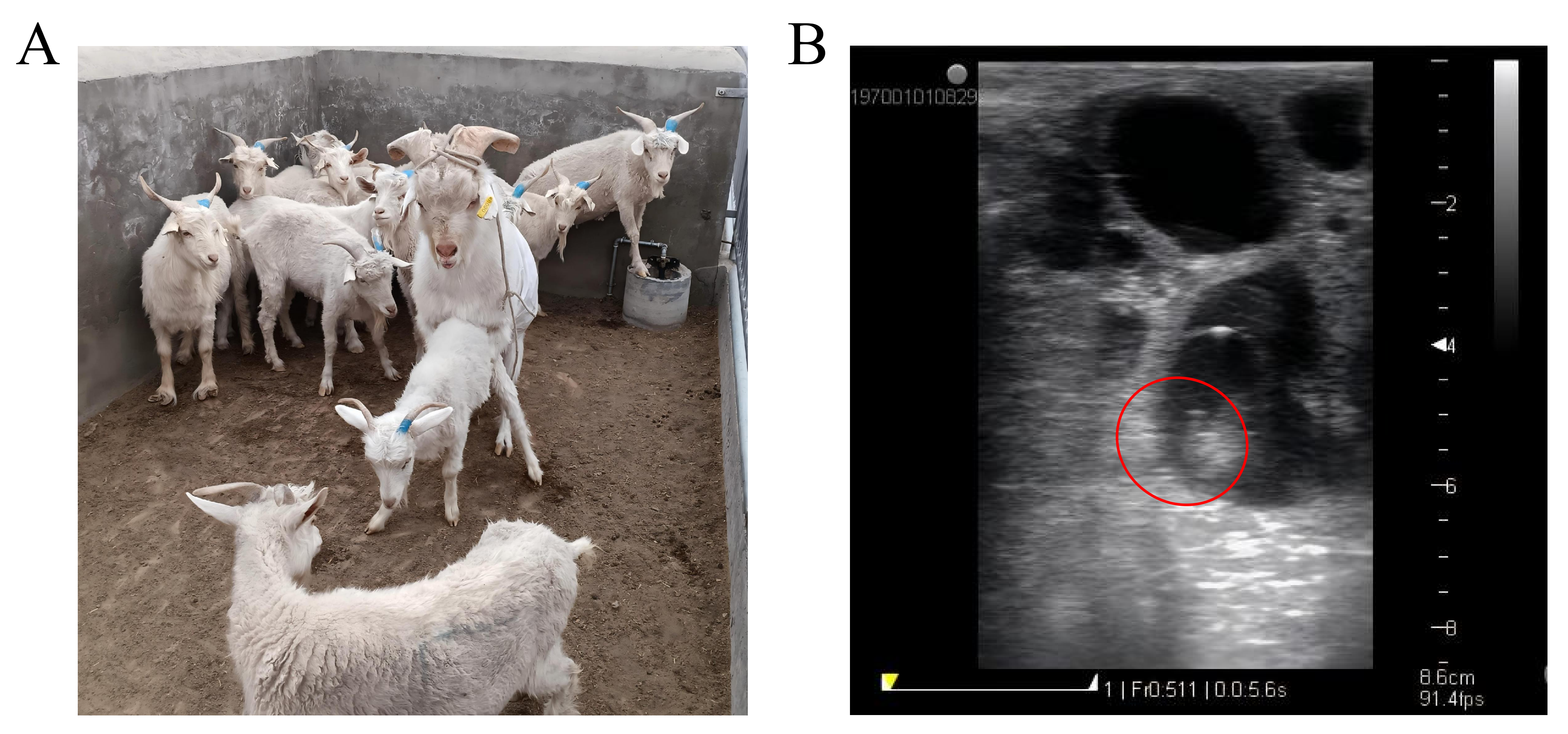

Supplement: SUPPLEMENTARY FIGURE 1 — Estrus detection and ultrasonic pregnancy diagnosis in does. (A) Does in estrus being mounted by a buck. (B) Pregnancy confirmed via ultrasonography 30 days after AI, with the early embryonic vesicle marked by a red circle. [file Image_1.tif]
